# Prevalence of Depression and Associated Factors among Diabetes Patients in East Shewa, Ethiopia: Bayesian Approach

**DOI:** 10.1155/2020/4071575

**Published:** 2020-10-21

**Authors:** Biruk Shalmeno Tusa, Mekuriaw Alemayehu, Adisu Birhanu Weldesenbet, Sewnet Adem Kebede, Getachew Asfaw Dagne

**Affiliations:** ^1^Department of Epidemiology and Biostatistics, College of Health and Medical Sciences, Haramaya University, Haramaya, Ethiopia; ^2^Department of Environmental Occupational Health and Safety, Institute of Public Health, College of Medicine and Health Sciences, University of Gondar, Gondar, Ethiopia; ^3^Department of Epidemiology and Biostatistics, Institute of Public Health, College of Medicine and Health Sciences, University of Gondar, Gondar, Ethiopia; ^4^College of Public Health, University of South Florida, Florida, USA

## Abstract

**Background:**

Depression is one of the most pressing public health problems and also highly prevalent comorbid condition among diabetes mellitus (DM) patients. Depression may impact lifestyle decisions and ability to poorly perform tasks which are risk factors for DM. For reducing the impact of depression among DM patients in developing countries, it is crucial to identify and assess associated risk factors of depression among DM patients, thereby designing effective management techniques. In line with this, the current study applies the Bayesian framework, which pools prior information and current data, to find factors associated with depression among DM patients.

**Methods:**

A hospital-based cross-sectional study was conducted at Adama Hospital and Medical College (AHMC) from March to April 2019. Data was entered into the Epi-data 3.1 then exported to the R software 3.4.4. Bayesian logistic regression models were fitted to the data using the Markov chain Monte Carlo (MCMC) algorithm. Estimates of model parameters including adjusted odds ratio (AOR) with 95% credible intervals (CI) were calculated.

**Results:**

A total of 359 adults with DM were included in the analysis. The prevalence of depression among diabetic patients was 9.22% (95% CI: 6.4% to 12.7%). Higher fasting blood sugar level (AOR = −1.012; HPD CI: (1.0020, 1.025)), having diabetic complication (AOR = 0.1876; HPD CI: (0.0214, 0.671)), history of hospital admission (AOR = 0.2865; HPD CI: (0.0711, 0.7318)), low medication adherence (AOR = 29.29; HPD CI: (3.383, 92.26)), and taking both insulin and oral antidiabetic medication (AOR = 24.46; HPD CI: (15.20, 49.37) were significantly and strongly associated with depression among DM patients.

**Conclusions:**

Prevalence of depression among diabetes patients in the catchment area of Adama Hospital, Ethiopia, was found to be very low. Higher fasting blood sugar level, diabetic complication, history of hospital admission, low medication adherence, and taking both insulin and oral antidiabetic medication were found to be strong predictors of prevalence of depression among DM patients. Based on the findings, we recommend that integrating screening and treating of depression, early detection and management of diabetic complication, and giving counseling to improve medication adherence is an effective approach for lowering the impact of depression on DM patients.

## 1. Introduction

Diabetes mellitus (DM) is a chronic disease which affects almost every organ in the human body. Globally prevalence of diabetes is increasing at an alarming rate affecting 463 million people. The World Health Organization (WHO) projected that 300 and 700 million people will suffer from diabetes by 2025 and 2045, respectively [1, 2]. The burden of diabetes is even higher in developing countries and in Ethiopia; systematic review result showed that prevalence of DM is between 2% and 6.5% [3].

Depression is one of the public health problems characterized by a state of low mood and aversion to activity that can affect a person's thoughts, behavior, feelings, and sense of wellbeing. It is one of the highly prevalent comorbid conditions among diabetic patients. Globally, depression is the second-leading cause of disability, and diabetic patients have been reported to be more likely to develop depression than nondiabetes people with estimated 15%-20% of people with diabetes struggling with moderate to severe form of depression [4–6].

Prevalence of depression among DM patients varies across countries with 8.3% % in the USA [7] to 71.8% in Iran [8], and in Ethiopia, it is ranging from 15.4 to 64.9% [9–15] with 39.73% pooled prevalence [16]. The presence of depression among diabetic patients increases the noncompliance to the medical treatment, decreases the quality of life, increases the risk of complication, results in poor prognosis, and increases mortality. Mortality from DM increases by 1.5-fold for patients with depression [17].

The occurrence of depression among diabetes is attributed to different factors. Studies indicated that burden of complications, financial stress, poor overall health status, knowledge of diabetes, poor social support and physical disability, and poor glycemic control are major factors associated with the presence of depression among DM patients [10, 18]. Smoking habit, increased number of comorbidities, higher level of cholesterol, and higher body mass index are also associated with depression among DM patients [19, 20].

Reducing the prevalence of depression among diabetes patients by designing effective management evidence on associated factors of depression among DM patients is crucial. For that reason, various studies have been piloted at different part of Ethiopia using classical logistic regressions with small datasets [9–15]. However, for small datasets, a Bayesian approach is preferred, and the current study applies the Bayesian framework (logistic regressions) which pools the prior information with current data to identify factors associated with depression among DM patients.

## 2. Methods

### 2.1. Study Design and Setting

A hospital-based cross-sectional study was conducted at Adama Hospital and Medical College (AHMC) from March to April 2019. The hospital, located in Adama city, Oromia National Regional State at 99 km to the southeast of Addis Ababa, the capital of Ethiopia. It has an outpatient department for chronic illness follow-up, and diabetes treatment is provided 2 days a week.

### 2.2. Sample Size Determination and Sampling Procedures

Sample size was calculated via the Open Epi software using a single population proportion formula by considering the following assumptions: prevalence of depression among diabetic patients at Felege Hiwot Referral Hospital, Bahir Dar, Northwest Ethiopia (*P* = 40.4%) [21], 95% confidence level, and 5% margin of error. The calculated sample size was 369, and by adding 10% nonresponse rate, the final sample size became 406. Patients diagnosed with DM who had follow-up for at least six months, age greater than 18 years, and visited the facility (AHMC) during the study period were selected as study participants.

A systematic random sampling method was used to select study participant. The sampling interval was computed by dividing the predictable number of diabetic patients per month into the sample size. The first study participant was selected by a lottery method from patients in the first sampling interval and turned out to be the second in the list, and then, every second person in the remaining sampling intervals was systematically selected until the desired sample size was reached. Details about the sampling method are available elsewhere [22].

### 2.3. Measurements and Operational Definition

Depression was the response variable that was measured using the Kessler 6 scales [23] which is validated in Ethiopia [24]. This instrument has 6 questions each asking the respondent how often they experienced symptoms during the past 30 days and containing 5-point Likert scales (1 = none of the time, 2 = a little of the time, 3 = some of the time, 4 = most of the times, 5 = all of the time). Then, item scores obtained from the scale were summed, and Serious Psychological Distress was considered when the score is 19 or more.

The explanatory variables included sociodemographic, behavioral (medication adherence and hazardous drinking habits), and clinical characteristics. Data related to sociodemographic and clinical factors were collected by using semistructure and pretested questionnaire which was developed by the principal investigator.

Medication adherence was measured using a 4-item Morisky medication adherence scale (MMAS). A high score indicates low levels of medication adherence [25]. Hazardous drinking was assessed using Fast Alcohol Screening Test (FAST). The Fast Alcohol Screening Test (FAST) is a short screening questionnaire for hazardous drinking comprising four questions. Then, item scores obtained from the scale were summed, and hazardous drinking was considered when the score is 3 or more [26].

Data was collected through face to face interview with document review by clinical nurses after receiving training on how to collect the data using both semistructured and standard questionnaire tools. Variables such as treatment modality (oral hypoglycemic agent, insulin therapy, and both oral hypoglycemic and insulin), diabetes-related complications, fasting blood sugar (records from the last visits were taken), and presence of documented comorbidity were obtained from patients' medical records. Initially, semistructured questionnaire was prepared in English version, then translated into Amharic and Oromiffa (local language) and again back translated to English by another person to check the consistency of the meaning.

### 2.4. Data Processing and Management

Each questionnaire was checked visually for completeness and consistency. Data was entered into the Epi-data 3.1 then exported to the R software 3.4.4. Finally, using the R software, the data was exported in text format in order to make it suitable for analysis in Win BUGS software [27]. Descriptive statistics and posterior summary statistics were presented and interpreted.

### 2.5. Statistical Analysis

#### 2.5.1. Likelihood Distribution

The outcome variable was the presence of depression which is typical Bernoulli and modeled via logistic regression using a Bayesian framework to find factors associated with the presence of depression among diabetic patients.

Yi and pi are the status (yes, no) and probability of the presence of depression, respectively, of all diabetic patients *j*. Assuming *Yj* has a Bernoulli distribution, *Yj* < Bernoulli (*pj*) and modeled covariates *Xj* and that is logit (*pj*) = XT *jβ* + *ϵ*, where *β* is the vector of regression coefficients.

#### 2.5.2. Specifying Prior Distributions for the Unknown Parameters

There are 25 unknown parameters (one intercept and 23 slope of the regression, *β*_0_, *β*_2_, *β*_3_, *β*_4_, ⋯, *β*_24_ and sigma). Let us use proper but noninformative prior; the priors on all parameters are assumed to be normal with mean zero and large variance or low precision (0, precision = 0.0001).

*Βj* ~ *N* (0, 0.0001), *j* ~ 0, 1 ⋯ 10.

#### 2.5.3. Posterior Distribution of the Parameters

Markov Chain Monte Carlo (MCMC) simulation was used to estimate the model parameters using Gibbs sampling in the WinBUGS software [28]. When the MCMC implementation was applied to the data (having a three-chain), convergence of the MCMC samples was assessed using standard tools within the WinBUGS software (kernel density, history plots, autocorrelation plots, and Gelman–Rubin convergence diagnostic). After discarding the initial 40,000 iterations as burn-in, a total of 807,003 iterations are used to obtain final samples of 19998 with thinning 50 to make estimation and final analysis. Estimates of model parameters including AOR with 95% credible intervals were calculated.

#### 2.5.4. Ethics Approval and Consent to Participate

Clearance was obtained from the institutional review board of the University of Gondar with reference number of IPH/180/06/2011. The purpose of the study was well explained, and informed consent was secured from study participants. No personal identifiers, such as name, address, and no private information, was collected.

## 3. Results

### 3.1. Sociodemographic Characteristics

A total of 359 adults with DM were included in the analysis with a response rate of 88.4%. The mean age of participants was 51 (±14.51) years with a range of 19 to 82 years. More than half (50.97%) of study participants were male. Near to one third (33.15%) of study participants attended primary education. Two hundred twenty-five (62.67%) of the participants were married, whereas 10 (2.79%) were divorced. Regarding occupation, 216 (60.17%) adults were unemployed, while 76 (21.17%) were employed as office workers (Table 1).

### 3.2. Behavioral and Clinical Characteristics

From the total of study participants, 22 (6.13%) were hazardous drinker. The mean (±SD) duration of living with DM was 10.38 (±5.27) years, and the mean (±SD) level of fasting blood sugar was 151.07 (±38.21) mg/dl. The majority of study subjects had type 2 diabetes (312 (86.91%)), 147 (40.95%) had comorbid disease other than DM, 83 (23.12%) had DM complication, more than half, 221 (61.56%) are on oral antidiabetic medications, and 101 (28.13%) had low medication adherence (Table 2).

### 3.3. Prevalence of Depression

The prevalence of depression among diabetes patients was 9.22% (95% CI: 6.4% to 12.7%). The highest prevalence of depression was observed among diabetes patients who are living in urban 32 (10.49%) and taking both oral and insulin medication 6 (21.43%). The prevalence of depression was also higher among diabetes patients who had a history of hospital admission 20 (19.42%), diabetic complication 14 (16.87%), and low medication adherence 21 (20.79%).

### 3.4. Factors Associated with Depression

To identify factors associated with depression among diabetes patients, we use a Bayesian logistic regression. Particularly, a Gibbs algorithm for all parameters was used to estimate the model parameters given in the Methods section. The convergences of the Gibbs algorithm were checked through MCMC assessment that involves checking the sequence, or Markov chain, for convergence and provides a representative sample from the posterior distribution.

### 3.5. Checking Convergence

#### 3.5.1. Time Series Plot

**Figure 1:**
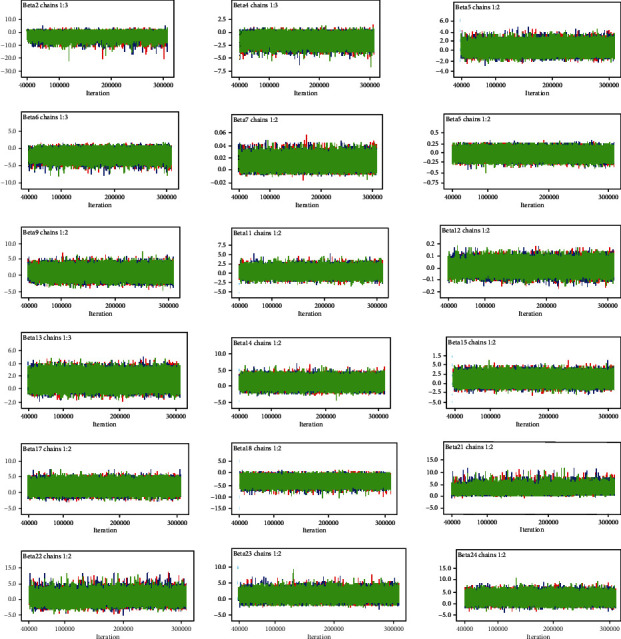
Time series for convergence of coefficients for the predictors.

According to Figure 1, the three independently generated MCMC chains are well mixed together or overlapped. Based on this, we can conclude that the simulation draws are reasonably converged, and therefore, we can be more confident about the accuracy of posterior inference.

#### 3.5.2. Density Plot

**Figure 2:**
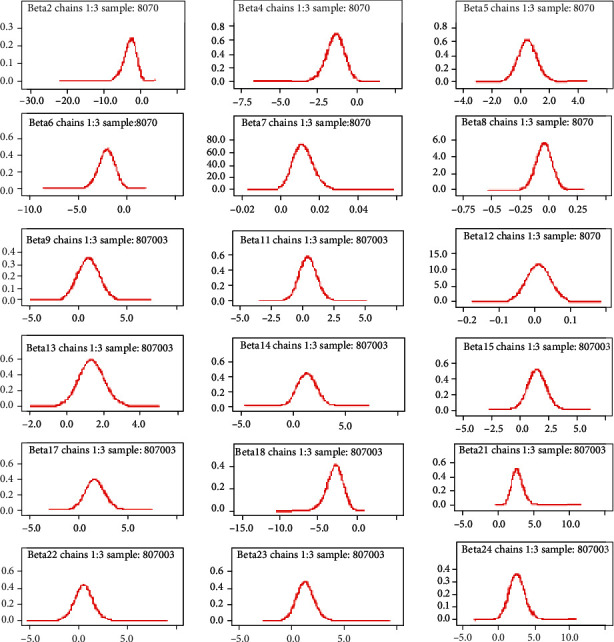
Density plot for convergence of coefficients for the predictors.

Density plot is one of the diagnostic plots that are used to check convergence in Bayesian analysis. As we can see from Figure 2, the plots for all parameters have unimodal densities, suggesting that the simulated parameter values were generated from stationary distributions.

#### 3.5.3. Autocorrelation Plot

**Figure 3:**
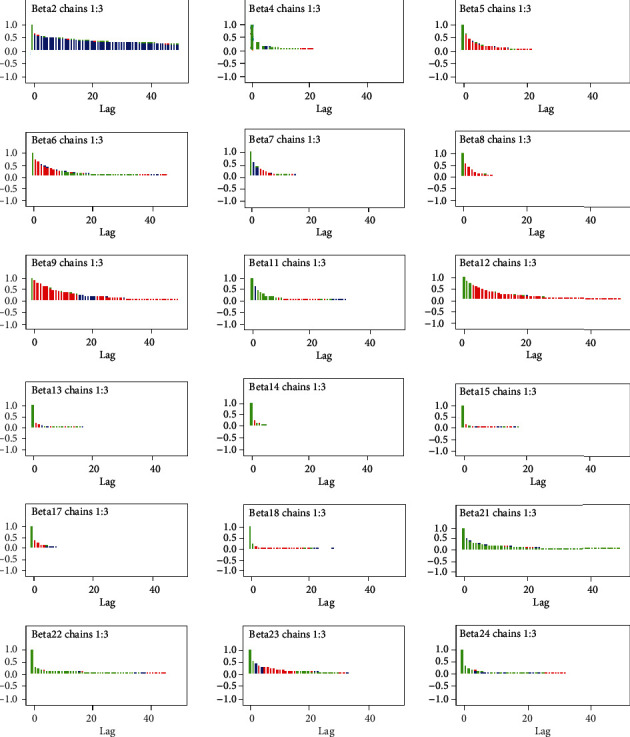
Autocorrelation plot for convergence of coefficients for the predictors.

Autocorrelation plot is another technique used to assess convergence in Bayesian analysis via MCMC. As we can observe from Figure 3 independent generated MCMC samples were attained after a thinning of 50 (50 lags).

#### 3.5.4. Gelman–Rubin Statistics

**Figure 4:**
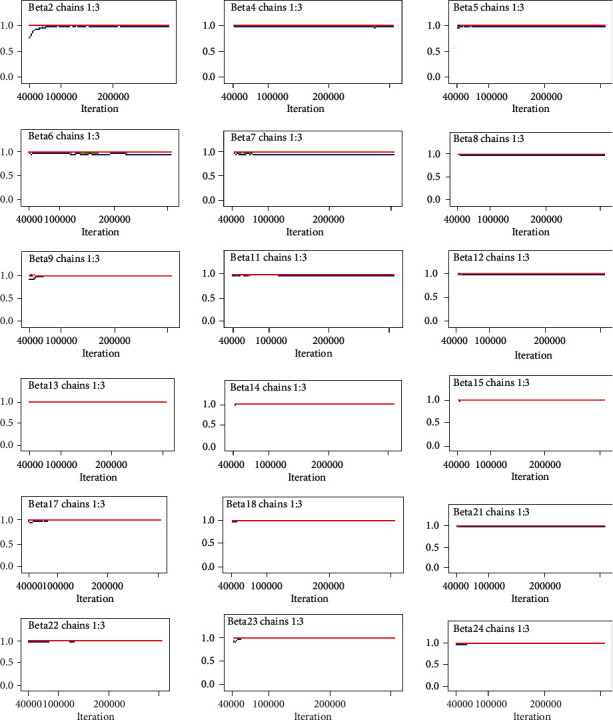
Gelman–Rubin statistic plot of for convergence of coefficients for the predictors.

Gelman–Rubin statistic is another method for assessing convergence. It can be applied only when multiple chains, based on different sets of initial values of parameters, are used. From the multiple chains, within and between variances are calculated, and if the ratio of the two is close to 1, then convergence is reached.  Figure 4 displays the Gelman–Rubin plots which clearly show convergence has been achieved.

#### 3.5.5. Assessing the Accuracy of Bayesian Logistic Model Fitting

The posterior summary estimates by the MCMC algorithm, especially by Gibbs sampler, have posterior mean, standard errors, Monte Carlo error, and credible intervals. If the MC error value for each parameter of interest is less than about 5% of its posterior standard error, then the posterior density estimates have accurate posterior estimates.

Accordingly, as we can see from Table 3, MC error for each significant predictor is less than 5% of its posterior standard deviation. This implies convergence and accuracy of posterior estimates are attained, and the model is appropriate to estimate posterior statistics. In view of the result of noninformative prior given in Table 3, considering the credible interval, “fasting blood sugar,” “DM complication,” “hospital admission,” “treatment regimen (both oral and insulin),” “marital status (widowed),” and “educational status (primary)” were significantly and strongly associated with depression among DM patients.

Marital status and educational status are sociodemographical parameters that have significant and strong association with depression among diabetes patients. Holding other variable constant, the odds of depression among widowed diabetic patients will be decreased by 91.47% (HPD CI: (0.0060, 0.3404)) as compared to married diabetic patients. Diabetes patients with primary level of education are 5.027 (HPD CI: (1.0680, 15.52)) times more likely to develop depression than diabetes patients with higher level education while keeping for all other variable constant.

As fasting blood sugar level increase by one unit, the odds of the depression will increase by 1.20% (HPD CI: (1.0020, 1.025)) while making other variable constant. Adjusting for other variables, the odds of depression among diabetes patients who had no diabetic complication is 81.24% (HPD CI: (0.0214, 0.671)) lower than their counterpart.

Keeping other variables constant, the odds of depression among diabetes patients who had no history of hospital admission in past one month is decreased by 71.35% (HPD CI: (0.0711, 0.7318)) as compared to those who had history of hospital admission. Low medication adherents' diabetes patients are 29.29 (HPD CI: (3.383, 92.26)) times more likely to develop depression than high medication adherents while adjusting for other variables.

Types of medication are one of the clinical factors that has a significant and strong association with depression among diabetes patients. Holding other variables constant, the odds of depression is increased by 24.46 times (HPD CI: (15.20, 49.37)) among diabetes patients who are taking both oral antidiabetic medication and insulin as compared to those are taking only oral antidiabetic medication.

## 4. Discussion

According to the current study, the prevalence of depression among diabetes patients was 9.22% (95% CI: 6.4% to 12.7%). This finding is comparable with studies conducted in Peru (11.2%) [19] and the USA (8.3%) [7]. However, this result is lower than studies conducted in Ethiopia (15.4 to 64.9%) [9–15], Tanzania (30%) [29], Nigeria (30%) [30], Uganda (34.8%) [31], Sudan (44%) [32], Pakistan (14.7%) [33], and Iran (71.8%) [8]. The possible explanation for these differences might be due to the types of diagnostic tools used to assess depression and its respective cut of value used to decide depression has occurred.

Various studies have documented that factors associated with depression among diabetes patients using the classical logistic regression. Risk factors were age [10, 15, 34], gender [14, 15, 35–38], marital status [15, 35, 37, 39], educational status [34, 37, 40], hazardous drinking [38, 39], medication adherence [13, 39], duration lived with DM [9, 14, 35], fasting blood sugar [41], DM complication [10, 12, 14, 36, 37], chronic illness other than DM [14, 34], and types of medication [34].

The present study also demonstrated factors associated with depression among diabetes patients at AHMC using a Bayesian logistic regression. Bayesian inference and frequentist (classical) statistics results are tough to compare; this is because they use different techniques with different tools for decision making. In frequentist (classical) statistics, standard deviation and confidence interval are used to make decision, while in Bayesian statistics, credible interval is used. However, findings from the Bayesian model are given preference, because the technique is more robust and precise than the traditional statistics.

The current study revealed that as fasting blood sugar level increase by one unit, the odd of the depression will increase by 1.20%. This finding can be explained by as blood glucose level increases, the diabetes patients manifesting like polyuria (excessive urination), polydipsia (excessive thirsty), polyphagia (excessive hunger), general weakness, and sleeping disturbances [42], which may lead to develop depression. This can also be defensible as those who have higher blood glucose want more health care services, are incapable to perform their daily activities, and are incompetent to join in different activities, which may lead to depression.

According to our study, the odds of depression among diabetes patients who had no diabetic complication is 81.24% lower than their counterparts. The possible explanation for this result might be, as diabetes patient develops diabetic-related complication, they need a considerable amount of time for healing, so they spend their times on clinic visits, hospitalization, and frequent ulcer dressings. The presence of a foot ulcer also creates anxiety due to a possibility of amputation.

The current study documented that low medication adherents' diabetes patients are 29.29 times more likely to develop depression than high medication adherents. The possible explanation might be good medications are essential in sustaining the optimal level of fasting blood sugar which reduces the chance of depression symptoms among diabetes patients.

According to the present study, the odds of depression is increased by 24.46 times among diabetes patients who are taking both oral antidiabetic medication and insulin than those who are taking only oral antidiabetic medication. This might be due to injection for insulin may cause discomfort, and these group diabetic patients (both oral antidiabetic medication and insulin) are more likely to have poor glycemic control this might eventually lead to develop depression symptoms.

The strength of the present study is using a Bayesian approach rather than classical approach to identify factors associated with depression among diabetes patients. This Bayesian method performs better in the sense of yielding larger coverage probabilities and smaller bias than the classic maximum likelihood approach. It also combined the prior information with present data.

However, this study has some limitations that should be kept in mind when interpreting the results. This study might be prone to social desirability bias since the data were collected through face to face interview. Finally, the study was conducted in a single hospital (AHMC) which limits the generalizability of the finding in Ethiopia.

## 5. Conclusion

This study documented that the prevalence of depression among diabetes patients in the catchment area of Adama Hospital, Ethiopia, was found to be very low. Higher fasting blood sugar level, diabetic complication, history of hospital admission, low medication adherence, and taking both insulin and oral antidiabetic medication were found to be strong predictors of prevalence of depression among diabetic patients. Based on the findings, we recommend integrating screening and treating of depression; early detection and management of diabetic complication and giving counseling to improve medication adherence is an effective approach for lowering the impact of depression on diabetes patients.

## Figures and Tables

**Table 1:** Sociodemographic characteristics of diabetes patients at Adama hospital and medical college, Adama city, East Shewa, Ethiopia 2019.

Variable	Frequency (*n* = 359)	Percentage (%)
Gender		
Female	176	49.03
Male	183	50.97
Residence		
Urban	306	85.24
Rural	53	14.76
Marital status		
Single	45	62.67
Married	225	75.21
Widowed	64	17.83
Separated	15	4.18
Divorced	10	2.79
Educational status		
Uneducated	60	16.71
Primary cycle	119	33.15
Secondary and above	180	50.14
Occupational status		
Unemployed	216	60.17
Employed	76	21.17
Other	67	18.66

**Table 2:** Behavioral and clinical characteristics of diabetic patients at Adama hospital and medical college, Adama city, East Shewa, Ethiopia 2019.

Variable	Frequency (*n* = 359)	Percentage (%)
Hazardous drinking		
Yes	22	6.13
No	337	93.87
Type of DM		
Type 1	47	13.09
Type 2	312	86.91
Types of medication		
Oral	221	61.56
Insulin	110	30.64
Both	28	7.80
Medication adherence		
Low	101	28.13
Medium	86	23.96
High	172	47.91
DM complication		
Yes	83	23.12
No	276	76.88
Chronic illness other than DM		
Yes	147	40.95
No	212	59.05
Hospital admission		
Yes	104	28.97
No	235	71.03

DM: diabetes mellitus.

**Table 3:** Summary statistics for the posterior distribution of model parameters.

Parameters (reference)	AOR	MC error	SD	HPD credible intervals	
				Lower (25%)	Upper (75%)
*β*_12_ (age)	1.0100	<0.0001	0.0350	0.9435	1.0810
Gender (male)					
*β*_11_ (female)	1.8990	0.0063	1.7790	0.3744	1.4530
Marital status (married)					
*β*_17_ (single)	9.7580	0.0468	15.640	0.8662	43.060
***β*_18_(widowed)**	**0.0853**	**<0.0001**	**0.0966**	**0.0060**	**0.3404**
*β*_19_ (separated)	9.477	0.2147	103.00	0.0001	53.430
*β*_20_ (divorced)	85.71	4.181	2468.0	0.7392	389.90
Educational status (secondary and above)					
***β***_**13**_**(primary)**	**5.027**	**0.0111**	**4.105**	**1.0680**	**15.52**
*β*_14_ (uneducated)	6.024	0.0190	8.509	0.6198	24.59
Occupational status (jobless)					
*β*_15_ (employed)	5.539	0.0170	5.713	0.8245	19.76
*β*_16_ (other)	0.4415	0.0023	0.6374	0.0001	2.090
Hazardous drinking (no)					
*β*_3_ (yes)	0.2282	0.0028	0.5028	0.0011	1.373
Medication adherence (high)					
***β*_21_(low)**	**29.29**	**1.103**	**405.4**	**3.383**	**92.26**
*β*_22 (_medium)	3.393	0.05873	22.02	0.2993	13.16
Duration lived with DM (*β*_8_)	0.9582	<0.0001	0.0685	0.8298	1.099
**Fasting blood sugar (** ***β*** **_7_**)	**1.012**	**<0.0001**	**0.0058**	**1.0020**	**1.025**
Type of DM (type 2)					
*β*_9_ (type 1)	5.677	0.0522	11.27	0.3278	27.67
**DM complication (yes)**					
***β*_6_(no)**	**0.1876**	**<0.0001**	**0.1838**	**0.0214**	**0.671**
Chronic illness other than DM (yes)					
*β*_5_ (no)	2.012	0.0068	1.6850	0.4530	6.127
**Hospital admission (yes)**					
***β*_4_(no)**	**0.2865**	**<0.0001**	**0.1746**	**0.0711**	**0.7318**
Types of medication (oral)					
*β*_22_ (insulin)	5.366	0.0457	16.69	0.7102	20.19
***β*_23_(both)**	**24.46**	**0.1659**	**81.47**	**15.20**	**49.37**

DM: diabetes mellitus; HPD: highest posterior density; MC: Monte Carlo; AOR: adjusted odds ratio; SD: standard deviation.

## Data Availability

The datasets supporting the conclusions of this article are available upon request to the corresponding author.

## Abbreviations

AHMC: Adama Hospital and Medical College
AOR: Adjusted odds ratio
CI: Credible interval
DM: Diabetes mellitus
FAST: Fast alcohol screening test
HPD: Highest posterior density
MC: Monte Carlo
MCMC: Markov chain Monte Carlo
MMAS: Medication adherence scale
SD: Standard deviation
WinBUGS: Win Bayesian using Gibbs sampling
WHO: World Health Organization (WHO).
